# New Genetic Biomarkers Predicting Azathioprine Blood Concentrations in Combination Therapy with 5-Aminosalicylic Acid

**DOI:** 10.1371/journal.pone.0095080

**Published:** 2014-04-24

**Authors:** Kazuhiko Uchiyama, Tomohisa Takagi, Yasunori Iwamoto, Norihiko Kondo, Tetsuya Okayama, Naohisa Yoshida, Kazuhiro Kamada, Kazuhiro Katada, Osamu Handa, Takeshi Ishikawa, Hiroaki Yasuda, Junichi Sakagami, Hideyuki Konishi, Nobuaki Yagi, Yuji Naito, Yoshito Itoh

**Affiliations:** Department of Gastroenterology and Hepatology, Kyoto Prefectural University of Medicine, Kyoto, Japan; Ospedale Pediatrico Bambino Gesu', Italy

## Abstract

**Background and Aims:**

Azathioprine (AZA) is widely used for the treatment of inflammatory bowel disease (IBD) patients. AZA is catabolized by thiopurine S-methyltransferase (TPMT), which exhibits genetic polymorphisms. It has also been reported that 5-aminosalicylic acid (5-ASA) inhibits TPMT activity, and that increased 6-thioguanine nucleotide (6-TGN, a metabolite of AZA) blood concentrations result in an increased number of ADRs. In this study, single nucleotide polymorphisms (SNPs) related to differential gene expression affecting AZA drug metabolism in combination therapy with 5-ASA were examined.

**Methods:**

To identify genetic biomarkers for the prediction of 6-TGN blood concentration, ExpressGenotyping analysis was used. ExpressGenotyping analysis is able to detect critical pharmacogenetic SNPs by analyzing drug-induced expression allelic imbalance (EAI) of premature RNA in HapMap lymphocytes. We collected blood samples on 38 patients with inflammatory bowel disease treated with AZA and corroboration of the obtained SNPs was attempted in clinical samples.

**Results:**

A large number of SNPs with AZA/5-ASA-induced EAI within the investigated HapMap lymphocytes was identified by ExpressGenotyping analysis. The respective SNPs were analyzed in IBD patients' blood samples. Among these SNPs, several that have not yet been described to be induced by AZA/5-ASA were found. SNPs within SLC38A9 showed a particular correlation with patients' 6-TGN blood concentrations.

**Conclusions:**

Based on these results, ExpressGenotyping analysis and genotyping of patients appears to be a useful way to identify inter-individual differences in drug responses and ADRs to AZA/5-ASA. This study provides helpful information on genetic biomarkers for optimized AZA/5-ASA treatment of IBD patients.

## Introduction

The immune-modulating thiopurines, 6-mercaptopurine (6-MP) and its pro-drug azathioprine (AZA), are widely used in inflammatory bowel disease (IBD) treatment [1]–[3]. AZA is a pro-drug of 6-MP carrying a methyl imidazole group attached to a sulfur carbon of 6-MP and metabolized to 6-MP by cleavage require the action of gluthatione S transferase. For the maintenance of remission in IBD, AZA and 6-MP are the first-line immunomodulators [4], [5]. Both AZA and 6-MP must be extensively metabolized before the pharmacologically active metabolites, 6-thioguaninenucleotides (6-TGN), are generated. The clinical efficacy of 6-MP has been reported to be correlated with erythrocyte levels of 6-TGN [6], [7]. The usefulness of monitoring 6-TGN blood concentrations in patients receiving AZA and 6-MP has also been suggested [8]–[10]. Although these thiopurine derivatives are considered to be relatively safe for maintenance therapy, several studies have reported discontinuation of thiopurine derivatives in up to 50% of patients during long-term therapy, mainly due to the development of adverse drug reactions (ADRs) [4], [11], [12]. AZA is catabolized by thiopurine S-methyltransferase (TPMT), which exhibits single nucleotide polymorphisms (SNPs). These SNPs have been reported to contribute to bone marrow suppression as ADRs [9], [13], [14]. Patients with mutant alleles are reported to exhibit lower enzymatic activity of TPMT and to be more likely to show bone marrow suppression. In the Japanese population, TPMT A719G is the most common allele [15]–[18] and is associated with low TPMT enzyme activity [19], [20]. However, in the Japanese population, the frequency of TPMT mutations is reported to be lower (2%) than that reported from Western countries [15]–[17], [21], [22]. It has also been reported that 5-aminosalicylic acid (5-ASA) inhibits TPMT activity, and that an increased 6-TGN blood concentration results in an increased number of ADRs [3]. However, the mechanism of ADRs induced by AZA has not yet been completely understood. Therefore, to identify new genetic biomarkers to predict 6-TGN blood concentrations before the administration of thiopurine is very valuable and important.

**Figure 1:**
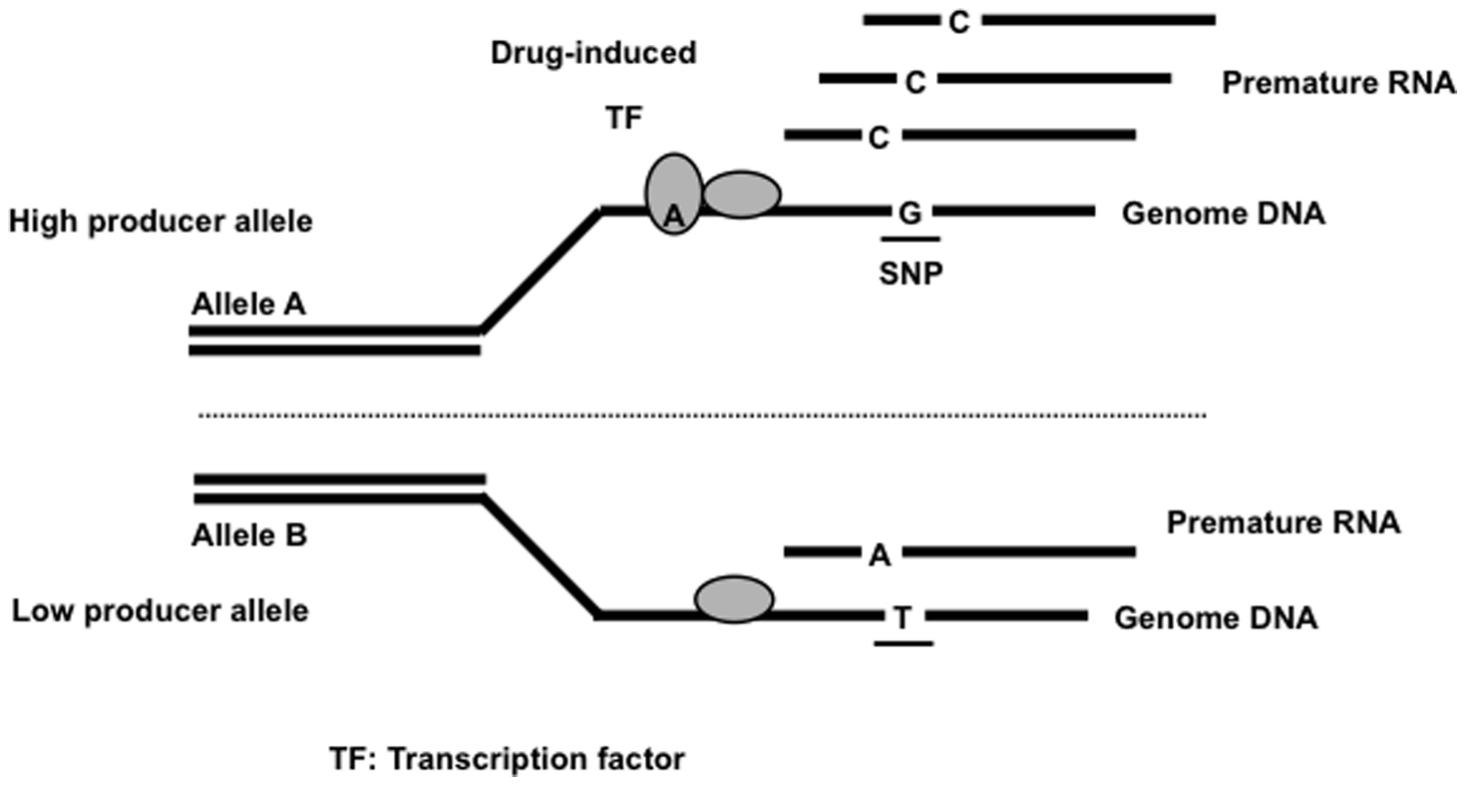
ExpressGenotyping analysis. ExpressGenotyping analysis can detect critical pharmacogenetic SNPs by analyzing drug-induced expression allelic imbalance (EAI) of premature RNA in HapMap lymphocytes. This new analysis technique has the potential to accelerate the realization of personalized medicine, which is closely associated with inter-individual differences in drug response and ADRs.

In this study, SNPs related to differential gene expression affecting AZA drug metabolism in AZA/5-ASA combination therapy were investigated. This genetic information is important to predict the 6-TGN blood concentrations of IBD patients treated with AZA/5-ASA. Recently, ExpressGenotyping analysis has been developed to explore the SNPs affecting gene expression changes of each allele (these gene expression changes are called Expression Allelic Imbalance (EAI)) after drug exposure (Figure 1). It is clear from a previous report that the concept of allelic variation such as this EAI is also important [23]–[25]. Such SNPs may be associated with inter-individual differences in drug response and ADRs. Therefore, ExpressGenotyping analysis can be used as a novel technology to detect critical genetic biomarkers that predict 6-TGN blood concentrations of IBD patients.

## Materials and Methods

### Patients

Patients with established IBD receiving AZA were enrolled between April 1^ST^ 2009 and August 31^ST^ 2009. All patients were Japanese persons attending the gastroenterology outpatient clinic at the Hospital of the Kyoto Prefectural University of Medicine. The characteristics of the enrolled patients are shown in Table 1. Of the 38 patients, 35 were also treated with 5-ASA. Clinical information related to drug administration for these patients is shown in Table 2. The protocol of this study was approved by the Ethics Committee of the Kyoto Prefectural University of Medicine and written informed consent was obtained from all patients before the original procedures were conducted. This study was performed in accordance with the ethical principles associated with the Declaration of Helsinki and registered in the University Hospital Medical Network Clinical Trials Registry (UMIN 000010197).

**Table 1:** Characteristics of enrolled patients.

Total (n)	38
Sex (n; male/female)	29/9
Age (years; median±range)	44.0±10.6
Disease (n; UC/CD/BD)	21/14/3
Location of UC patients (n = 21)	
Pancolitis	19
Distal colitis	2
Location of CD patients (n = 14)	
Colon	4
Colon and ileum	6
Ileum	4
Duration of IBD (years; median±range)	12.8±8.97

UC: ulcerative colitis, CD: Crohn's disease, BD: Behcet's disease.

**Table 2:** Clinical information of drug administration for patients.

Sample number	Catalog ID	Dose of azathioprine (mg/day)	Dose of 5-ASA (mg/day)	Concentration of 6-TGN (pmol/8×10^8^ RBCs)	Risk ratios (6-TGN/AZA)
1	5842535	75	3600	195	2.60
2	5779006	50	2250	474	9.48
3	770916	25	1500	423	16.92
4	4872959	25	3000	130	5.20
5	5459355	50	3000	287	5.74
6	6232762	50	2250	243	4.86
7	2639084	50	2250	164	3.28
8	0156158	50	1500	144	2.88
9	5571916	50	3000	50	1.00
10	3281892	25	2250	464	18.56
11	2852065	100	3000	353	3.53
12	162980	50	2250	572	11.44
13	0777935	100	0	253	2.53
14	5445356	100	2250	178	1.78
15	6007418	50	3600	152	3.04
16	5294675	25	2250	144	5.76
17	4549910	100	3000	87	0.87
18	5999831	25	2400	163	6.52
19	2002116	25	2400	54	2.16
20	6158273	50	4000	365	7.30
21	6176417	50	3600	293	5.86
22	4255178	50	3600	557	11.14
23	6230023	50	3600	99	1.98
24	0185310	75	2000	595	7.93
25	0437466	50	3000	488	9.76
26	1830237	50	2250	359	7.18
27	1996388	50	3000	146	2.92
28	2240525	50	1500	301	6.02
29	2366881	50	3000	287	5.74
30	4782476	50	2000	193	3.86
31	4916624	50	3000	201	4.02
32	5478242	100	2250	380	3.80
33	5480939	25	3000	292	11.68
34	5615377	100	4000	113	1.13
35	5796199	100	0	50	0.50
36	5892802	75	3000	366	4.88
37	5906785	50	2000	561	11.22
38	6176174	50	0	288	5.76

Clinical information related to drug administration for patients.

Since the 5-ASA dose was less than 2000 mg/day in sample numbers 3, 8, 13, 28, 35, and 38, they were not used for data analysis in this study.

### Measurement of 6-TGN

The blood 6-TGN concentration was measured by Mitsubishi Chemical Medience Corporation using HPLC (Inertsil ODS-3 (3.0×100 mm, 4 µm). This is the modified method from the previous report [23].

### Exposure of HapMap lymphocytes to drugs

AZA and 5-aminosalicylic acid (5-ASA) were obtained from MP Biomedicals, Inc. (Tokyo, Japan). They were dissolved in RPMI medium 1640 (Invitrogen, Van Allen Way Carlsbad, CA, USA) including 15% fetal bovine serum (Invitrogen) as vehicle for in vitro experiments. Suspensions of 30 different HapMap lymphocytes from Japanese subjects (Coriell, Camden, NJ, USA), at a concentration of 2.5×10^6^ cells in 0.5 mL of RPMI medium 1640 (Invitrogen) including 15% fetal bovine serum, were incubated with 4.5 mL of AZA/5-ASA (100 µM AZA and 10 mM 5-ASA). The suspensions were sampled after 24 hours and used for the following assay. As controls, the respective HapMap lymphocytes were treated in the same way, but only with the vehicle RPMI medium 1640 including 15% fetal bovine serum (no drugs).

### mRNA expression analysis

Total RNA was extracted from the AZA/5-ASA-treated and control cells using TRIzol Reagent (Invitrogen). Total RNA concentration was determined using a NanoDrop ND-1000 instrument (NanoDrop Technologies, Wilmington, DE, USA), and RNA quality was checked with an Agilent 2100 Bioanalyzer (Agilent Technologies, Santa Clara, CA, USA). Starting with 250 ng of total RNA, GeneChip U133 Plus 2.0 Array analysis (Affymetrix, Santa Clara, CA, USA) was conducted following the manufacturer's protocol, using the GeneChip 3′IVT Express Kit (Affymetrix) and the GeneChip Hybridization Wash and Stain Kit (Affymetrix). The raw expression signal obtained through a GeneChip Instrument System (Affymetrix) was normalized by scaling of the target signal to 100. For further data processing, probes were selected showing a P call in the detection step of the U133 Plus 2.0 Array among these selected probes; any probe set showing a ≥2-fold increase or decrease (AZA/5-ASA- treated vs. vehicle) of gene expression for a given sample was considered a significant change.

### SNP 6.0 array analysis

DNA was extracted from the 30 different untreated HapMap lymphocytes using a QIAamp DNA mini Kit (QIAGEN), and the quality and concentration of the DNA samples were determined with a NanoDrop ND-1000 (NanoDrop Technologies). On the other hand, DNA of IBD patients was extracted with FALCO Biosystems (Kyoto, Japan). Starting with 500 ng of DNA, the 6.0 SNP Mapping Array assay was performed using the GeneChip 6.0 SNP Mapping Assay Kit (Affymetrix) following the manufacturer's protocol. Data for SNP typing were determined using the Genotyping Analysis Software (GTYPE) Version 4.1 of the GeneChip Instrument System (Affymetrix). Based on the 6.0 SNP Mapping Array genotyping information, an AA, AB, or BB call was obtained. In this analysis, a Birdseed v1 or v2 algorithm was included in the calculation [26].

### MassARRAY analysis

DNA of IBD patients was extracted with FALCO Biosystems. Genotyping of SNPs (rs1142345, rs1800584, rs56161402, rs6921269, rs2842951, rs2842934, rs1800460, rs2518463, rs72552739, rs1800462, and rs3898137) in the TPMT gene was performed using the Sequenom MassARRAY technology platform (Sequenom, San Diego, CA, USA), with the iPLEX Gold Reagent Kit (Sequenom), SpectroCHIP ARRAYS, Clean Resin Kit (Sequenom), and HotStarTaq DNA Polymerase (QIAGEN, Hilden, Germany) based on the single-base extension reaction. Locus-specific PCR primers and allele-specific detection primers were designed using the MassARRAY Assay Design 3.1 software (Sequenom). Allele detection was performed using a MassARRAY Analyzer Compact MALDI-TOF mass spectrometer (Sequenom), a MassARRAY Nanodispenser (Sequenom), and the MassARRAY TYPER 3.4 software (Sequenom).

### Correlation analysis of SNPs and 6-TGN risk ratios in IBD patients

We difined the risk ratio as the proportion of blood 6-TGN concentration and the dose of AZA intake (6-TGN concentration/AZA dose). The correlations between the AA, AB, and BB genotypes and 6-TGN risk ratios of clinical data in IBD patients were analyzed. For the correlation analysis, the genotypes of each sample from 32 individuals were plotted on the X-axis as AA = 0, AB = 1, and BB = 2, and 6-TGN risk ratios for the corresponding genes were plotted on the Y-axis. These for which all AA, AB, and BB genotypes were observed in at least 2 samples were selected for subsequent analysis, and r^2^ was calculated for the combination of each SNP and 6-TGN risk ratio. With this correlation analysis, SNPs with r^2^≥0.4 (p-values<0.01) with 6-TGN risk ratios were identified, and these were considered genetic biomarkers.

### ExpressGenotyping analysis

Premature RNA expression analysis was performed using the ExpressGenotyping reactor and ExpressGenotyping analyzer (Japanese Patent Number: 4111985, Method for identifying a gene with allelic variation in gene expression (FILING DATA: December 15, 2005)). This new analysis technique is performed using the GIM algorithm [27]–[29]. The ExpressGenotyping reactor consists of a group of reaction steps where cDNA is synthesized preferentially from the premature RNA in the total RNA. The subsequent assay using the synthesized cDNA is essentially similar to the Affymetrix 6.0 SNP Mapping Array's assay, but instead of ordinary DNA typing, quantitative allele-specific expression information is obtained. The ExpressGenotyping analyzer combines the information from the DNA and cDNA typing and detects variations of premature RNA expression from each allele at particular SNPs. Consequently, comprehensive premature RNA expression assays using the ExpressGenotyping reactor and ExpressGenotyping analyzer can detect genes with differential allelic expressions.

### Detection of drug-induced EAI by ExpressGenotyping analysis

To identify genetic biomarkers involved in the AZA/5-ASA-response, ExpressGenotyping analysis was performed as follows: 1) The ExpressGenotyping data outputs of drug-treated and vehicle-treated cells were obtained as a text file; 2) The respective vehicle and drugs files were combined with probe ID; 3) Probes with a heterozygous (AB) call within a gene region (3UTR, 5UTR, CDS, exon, and intron) were selected; 4) The expression ratios of premature RNA (vehicle sample (A allele/B allele or B allele/A allele) “vehicle EAI (max)”, drug sample (A allele/B allele or B allele/A allele) “drug EAI (max)” were calculated; 5) SNPs with an EAI ratio [calculated as drug EAI (max)/vehicle EAI (max)] of ≥2 were considered to show an EAI specific for drug exposure; and 6) EAI data of 30 test samples that were cell lines were collected. Next, AB heterozygous genotype analysis based on statistics was performed to identify responsive SNPs from the cells exhibiting the AB heterozygous genotype. SNPs with 3 or more heterozygous calls among the 30 test samples were used for this analysis. In this method, SNPs showing p-values<0.01 in either *analysis of covariance* of expression values or the *t*-test of the difference of inter-allelic expression ratios in cells under stimulation with test compounds and also exhibiting inter-allelic expression ratios of ≥2-fold were added to the list of test compound-responsive SNPs.

### Comparative analysis between clinical and ExpressGenotyping data

The genetic biomarkers analyzed in IBD patients' blood samples were correlated with drug-induced EAI obtained by ExpressGenotyping analysis. In this analysis, a coincidence between genetic biomarkers of clinical data (r^2^ = 0.4 or more, p-value = 0.01 or less) of IBD patients and drug-induced EAI values of ≥2 was detected based on the statistics of the AB heterozygous genotype analysis.

### Statistics

Risk ratio was assessed for each genotype group of patients. The data were compared using correlation analysis and *t* test. Prizm version 5.0 d (GraphPad Software Inc, La Jolla, CA) and SciPy (statistical library of Python) (http://www.scipy.org/) were used for all statistical analysis. All statistical significance was defined as *P*<.05.

## Results

### 6-TGN blood concentration and 5-ASA dose

**Figure 2:**
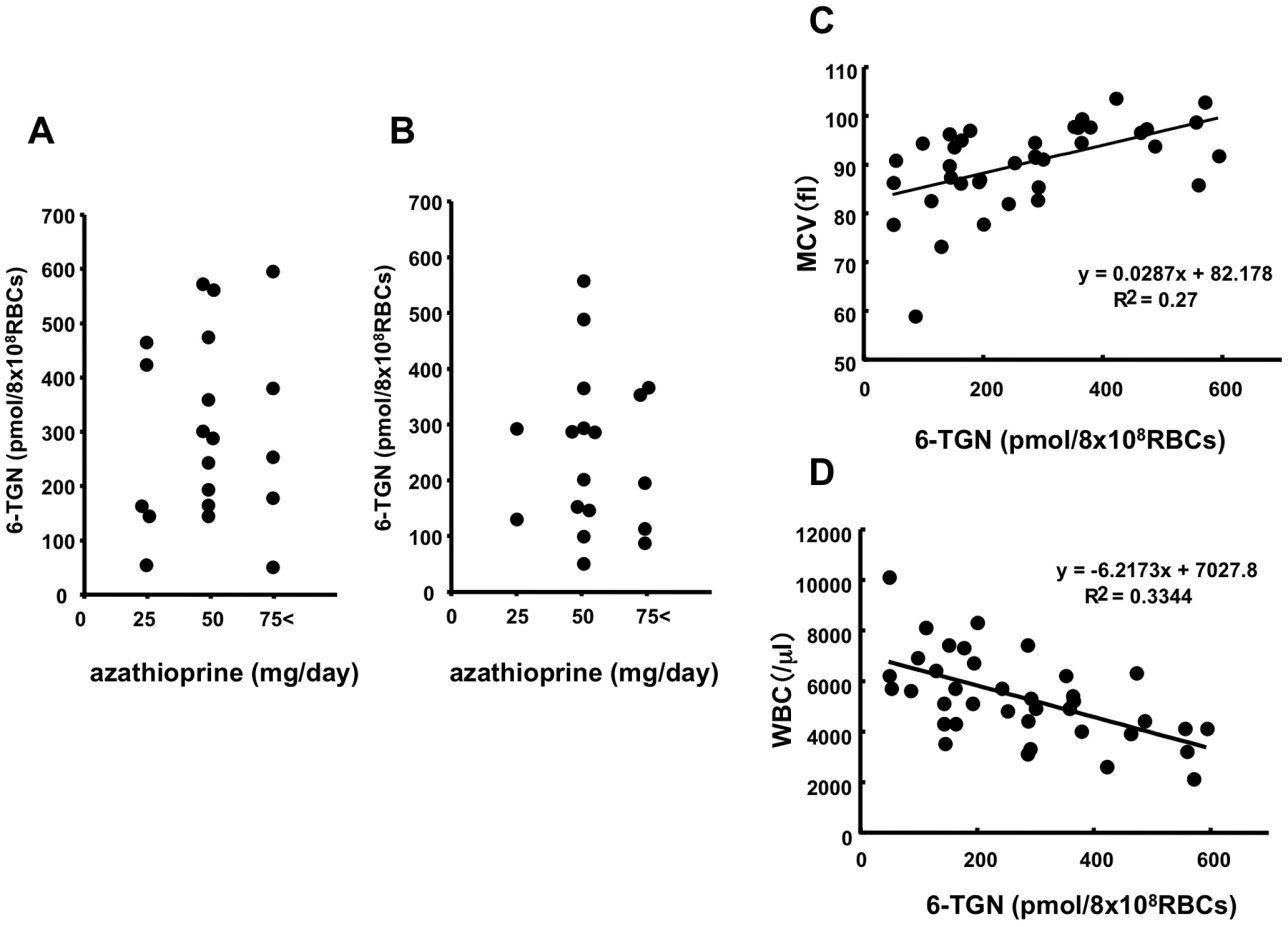
Frequency distribution of 6-TGN levels with the treatment of AZA and 5-ASA. (A) 6-TGN levels of patients taking less than 3000 mg/day of 5-ASA. (B) 6-TGN levels of patients taking more than 3000 mg/day of 5-ASA. Relationships between 6-TGN levels and MCV (C) and between 6-TGN levels and WBC (D).

The relationship between 6-TGN blood concentration and the 5-ASA dose was analyzed. There was no positive correlation between 6-TGN blood concentration and low-dose 5-ASA (Figure 2a) or high-dose 5-ASA (Figure 2b) administration.

As shown in Figure 2c, mean corpuscular volume (MCV) was highly correlated with 6-TGN blood concentration. The white blood cell (WBC) level showed a significantly negative correlation with 6-TGN levels (Figure 2d).

### mRNA expression analysis

Gene expression changes of mRNA were observed in mRNA expression analysis of HapMap lymphocytes from Japanese subjects. The scatter plots (Figure S1) showed significant changes in mRNA expression for the 100 µM AZA and 10 mM 5-ASA-treated vs. vehicle-treated HapMap lymphocytes for 24 hours. As the probe numbers of gene expression changed, by observing an average of 1257 up (change ≥2×)-regulated-probes (796 genes) and 815 down (change ≤0.5×)-regulated-probes (621 genes), 100 µM AZA and 10 mM 5-ASA treatment caused a marked effect on mRNA expression. GOTERM analysis of the Database by Annotation Visualization and Integrated Discovery (DAVID) showed the up-related terms (apoptosis, programmed cell death, and death etc.) and the down-related-terms (M phase, cell cycle phase, and cell cycle etc.) (http://david.abcc.ncifcrf.gov/). Based on the above results, exposure with 100 µM AZA and 10 mM 5-ASA for 24 hours was selected as the experimental condition for the subsequent ExpressGenotyping analysis of 30 different HapMap lymphocytes (Figure S2).

### SNPs analysis in IBD patients

**Figure 3:**
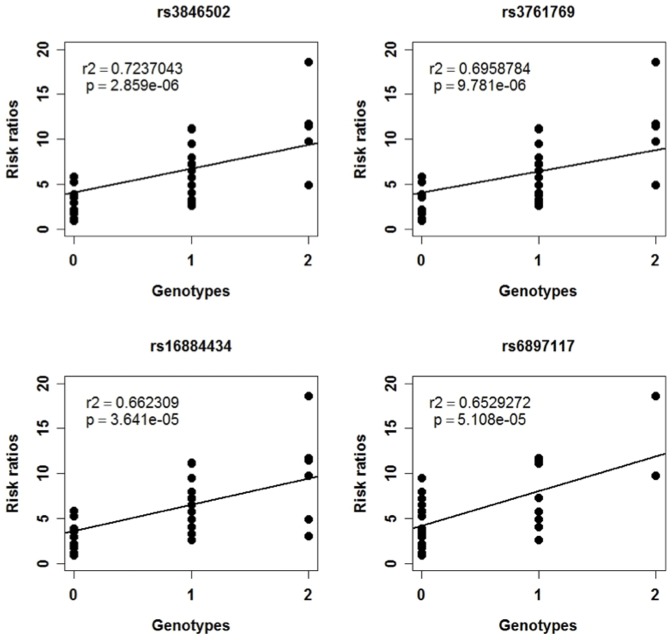
SLC38A9 SNPs of IBD patients. SLC38A9 SNPs were determined by correlation analysis with genotypes and 6-TGN risk ratios in IBD patients.

Correlation analysis of SNPs and 6-TGN risk ratios in IBD patients with SNP 6.0 Array analysis detected 1286 SNPs (516 genes) with r≥0.4 (p-values<0.01) (Figure S3). The GOTERM analysis of the database by DAVID identified cell adhesion, biological adhesion, and cell projection morphogenesis, etc. The top most significant SNPs were genotypes for rs3846502, rs3761769, rs16884434, and rs6897117 in SLC38A9 (Figure 3). SLC38A9 has been reported as a transporter gene. On the other hand, no correlations with SNPs in TPMT gene were obtained. Therefore, in order to perform a more detailed analysis of the TPMT gene, by MassARRAY analysis, the genotypes (AA, AB, or BB) of various TPMT SNPs were identified. TPMT is an enzyme that metabolizes AZA to 6-TGN, and some SNPs cause differences in metabolic activity. Four SNPs of TPMT have been analyzed (rs1800462, TPMT*2 [30]–[32]; rs1800460, TPMT*3B [33]; rs1142345 “A719G”, TPMT*3C [34]; rs1800584, TPMT*4 [35]) that affect 6-TGN blood concentration and, therefore, hematological toxicity. No difference in the distribution of SNPs was found, and genotype effects of the risk allele on hematological toxicity were not seen in all clinical samples measured (Figure S4). Other taq SNPs (rs56161402, rs6921269, rs2842951, rs2842934, rs2518463, rs72552739 and rs3898137) in TPMT showed no differences in these genotypes, as with the SNPs noted above. These SNPs of rs2842951, rs2518463, and rs3898137 observed in different distributions did not show significant correlations (rs2842951 (r = 0.08014, p-values = 0.66281), rs2518463 (r = 0.10855, p-values = 0.55427), and rs3898137 (r = 0.04124, p-values = 0.82268)). From the above results, differences in the 6-TGN risk ratios were found to not be effected by TPMT SNPs of IBD patients.

### Detection of drug-induced EAI

**Figure 4:**
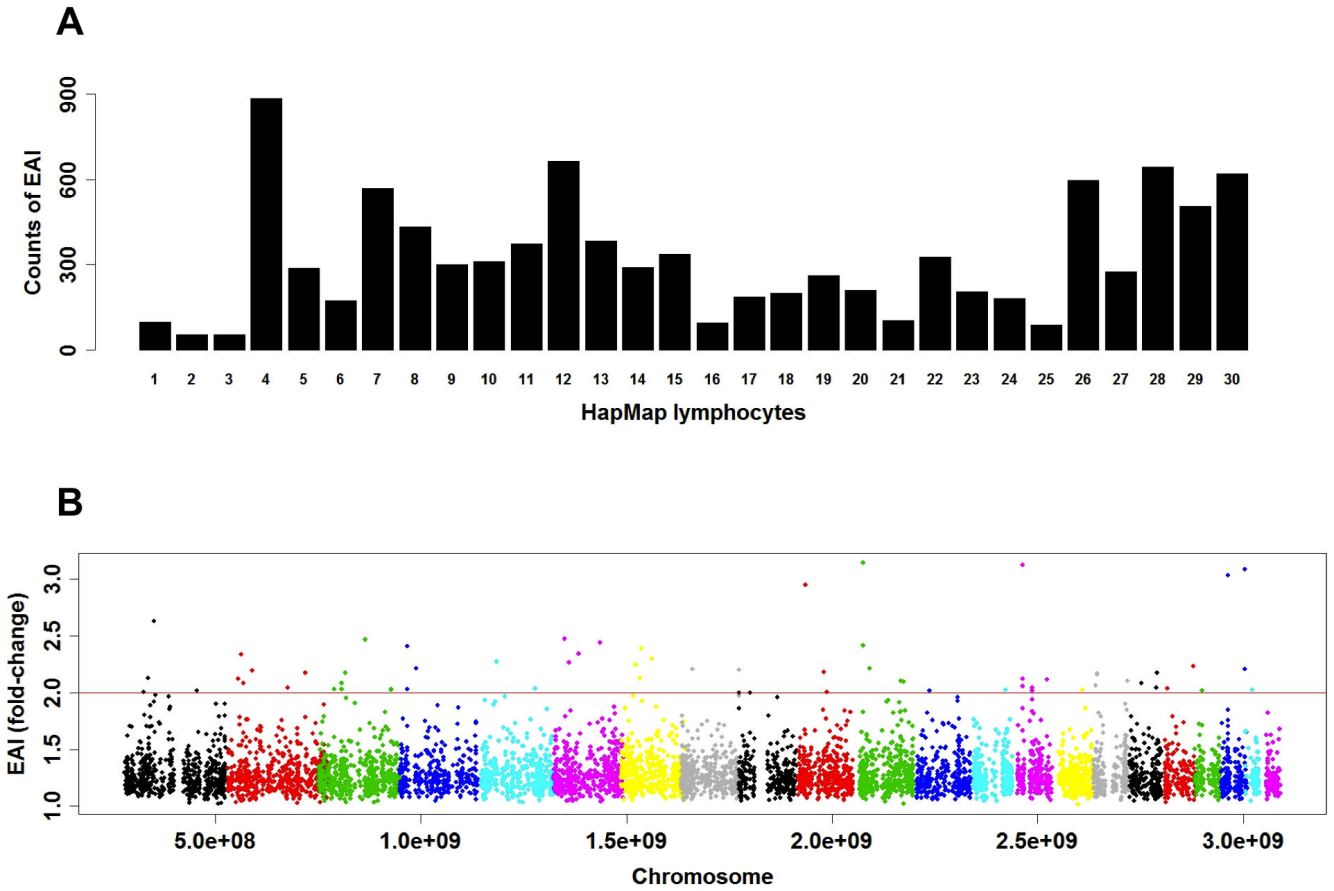
Number of drug-induced EAIs for 30 different HapMap lymphocytes by ExpressGenotyping analysis. (A) For the drug-induced EAIs, SNPs with an EAI ratio ≥2 were selected. Dispersion diagram of drug-induced EAIs for each chromosome. (B) The drug-induced EAI of fold-change in the dispersion diagram detected with AB heterozygous genotype analysis by ExpressGenotyping. Details of the AB heterozygous genotype analysis are described below. SNPs with the AB heterozygous genotype in >3 of 30 HapMap lymphocytes were used to search for SNPs responsible for producing the drug-induced intracellular allelic imbalance. These analyses were performed using the SNPs for which the AB heterozygous genotype was observed in at least 3 samples.

For the detection of AZA/5-ASA-induced EAIs, 8373 SNPs (3338 genes) with an EAI ratio ≥2 in ExpressGenotyping analysis (Figuer S5) were selected, and the numbers of AZA/5-ASA-induced EAIs for each HapMap lymphocyte are shown in Figure 4a. The dispersion diagram of the AZA/5-ASA-induced EAIs in the AB heterozygous genotype analysis for each chromosome is shown in Figure 4b. The most significant genes showing ≥3 fold-change of EAI were the SNPs of chromosome 11: GALNTL4 (rs2129523), chromosome 14: NPAS3 (rs12433161), chromosome 20: PHACTR3 (rs6015561) and chromosome 20: MACROD2 (rs8116068). In addition, the AB heterozygous genotype analysis of ExpressGenotyping data revealed reliable AZA/5-ASA-induced EAIs (64 SNPs (55 genes). The results are shown in (Figure S6). GOTERM analysis of the database with DAVID identified the regulation of apoptosis, regulation of programmed cell death, and regulation of cell death, etc. in the AB heterozygous genotype analysis. These EAI-related SNPs on ExpressGenotyping analysis may be important as genetic biomarkers affecting gene expression changes of each allele in predicting 6-TGN blood concentrations in patients taking AZA/5-ASA medication.

### Comparative data analysis by clinical data and ExpressGenotyping analysis

**Figure 5:**
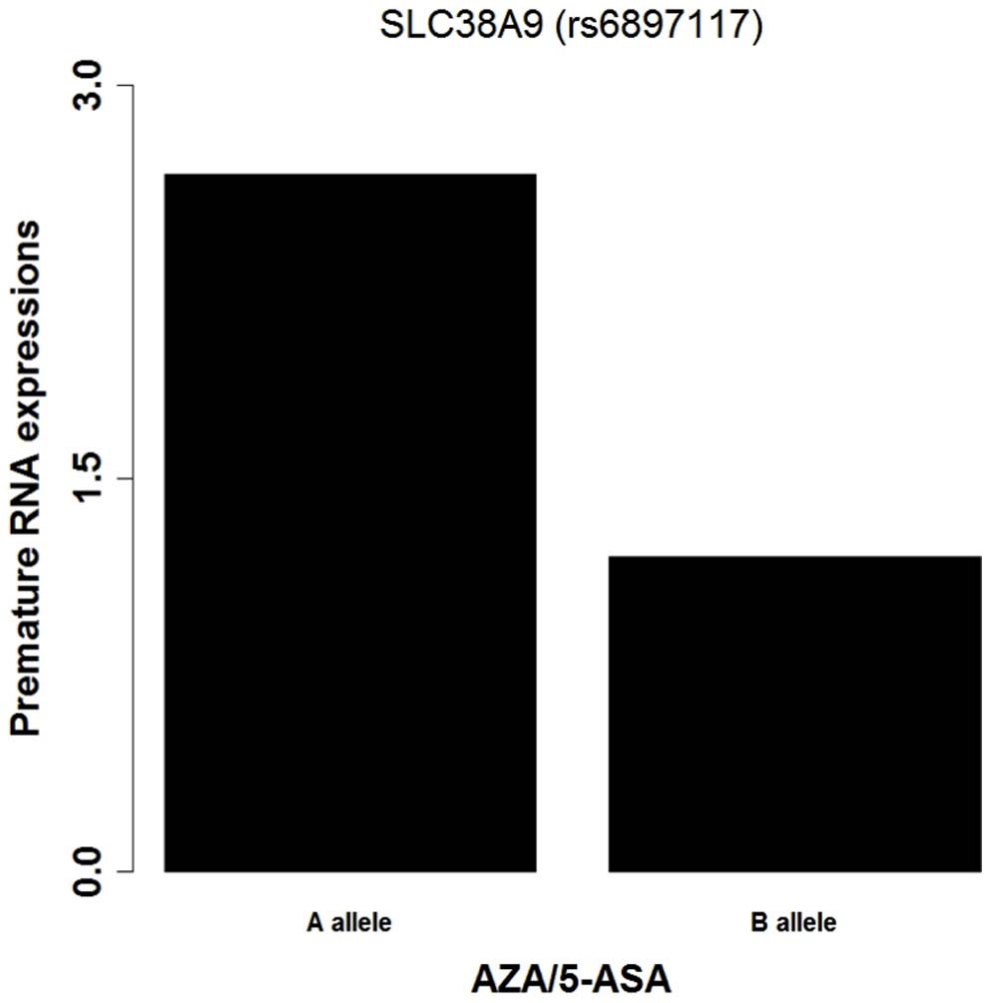
Results of drug-induced EAI for SNP (rs6897117) of SLC38A9. The SNP of SLC38A9 shows a consistent relationship between 6-TGN risk ratios and drug-induced EAI.

An attempt was made to identufy genetic biomarkers by combining 6-TGN risk ratios of clinical data and AZA/5-ASA-induced EAI of ExpressGenotyping analysis. In this comparative data analysis, SNPs in SLC38A9 showed a correlation with the 6-TGN risk ratios and AZA/5-ASA-induced EAI. The SNP rs6897117 of SLC38A9 was significant in the clinical data, and ExpressGenotyping analysis showed an EAI ratio ≥2 in GM18975 (Figure 5). The EAI ratio of SLC38A9 showed that the gene expression level of allele B was reduced, and it may increase the 6-TGN risk ratios. Therefore, these SNPs identified by ExpressGenotyping analysis might serve as genetic biomarkers for prediction of 6-TGN blood concentrations in patients taking AZA/5-ASA medication.

## Discussion

In the present study, new SNPs on SLC38A9 as genetic biomarkers to predict blood 6-TGN concentrations in patients taking AZA were identified. As the usage of AZA for IBD patients is now increasing, and the efficacy of AZA to maintain remission has been reported to be superb, it is very important that complications do not occur with AZA. In the analysis of TPMT SNPs of IBD patients in this study, the difference in the 6-TGN risk ratios was found to not have the risk allele related to hematological toxicity. Compared to the previous genetic biomarker, the new biomarker, SNPs of SLC38A9, was more sensitive.

In this study, 6-TGN concentration was not correlated with the dose of AZA treatment. Most of the studies have also reported no relationship between AZA dose and 6-TGN concentration [36]. This means that weight-based dosing of AZA does not guarantee therapeutic metabolite concentrations, and the efficacy of AZA metabolism might determine 6-TGN concentrations. As the number of WBCs and 6-TGN concentration showed a reverse correlation in the present study, prediction of blood 6-TGN concentration before AZA treatment will contribute to prevent leukopenia induced by 6-TGN.

Solute carriers (SLCs) are the largest group of transporters in the human genome [37], [38]. Many of the SLCs have important functions in cellular processes, including roles as transporters for amino acids and neurotransmitter cycling, while others are passive transporters or exchangers [38]–[40]. The SLCs have been categorized into at least 46 different families with varied biochemical properties [41], and two more families have recently been identified [42]. In mammals, the SLC proteins form four major phylogenetic groups, α-, β-, γ-, and δ-groups, with proteins in each group having a common evolutionary origin. The β-group includes the SLC32, SLC36, and SCL38 families, and it is the second largest phylogenetic cluster of amino acid transporters.

The SLC38 family consists of 11 members. The orphans SLC38A7-11 were only recently identified, and their substrate of transport has thus far not been characterized [43], while the other members, SLC38A1-6, are functionally classified as sodium-coupled neutral amino acid transporters, also known as SNATs. SNAT1 (SLC38A1, [44], [45]), SNAT2 (SLC38A2, [46]–[49]), and SNAT4 (SLC38A4, [50], [51]) have been further classified into system A, while SNAT3 (SLC38A3) and SNAT5 (SLC38A5) belong to system N transporters [52]–[54]. The system N/A classification depends on the functional properties and patterns of substrate recognition. SNAT6 (SLC38A6) [55] and the other more recently discovered members of the family have not been classified according to the N/A system. All SNAT genes, except SNAT4 [50], are expressed in the brain, with SNAT1 [44], [56] and SNAT2 [57] expressed both in astrocytes and neurons, while SNAT3 [52] and SNAT5 [58] are located in glial cells. No exact cellular localization of the protein or substrate profile has been reported for the orphan members of the SLC38 family. L-glutamine is a favored substrate for most of the members throughout the SLC38 family, and it is suggested that the transporters are part of the glutamine-glutamate cycle in the CNS [52], [54]. In this study, the genotype of SLC38A9 was found to be related to the blood 6-TGN concentration. The direct effect of SLC38A9 SNPs on 6-TGN blood concentration is still unclear, and further study will be necessary to reveal the pathway from SLC38A9 SNPs to the metabolism of AZA.

ExpressGenotyping analysis developed by HaploPharma Inc. is an innovative technology to detect critical pharmacogenetic SNPs and to provide genetic biomarkers to analyze them. ExpressGenotyping analysis enables the discovery of genetic biomarkers with a much smaller number of samples than with conventional approaches, such as genome-wide association study (GWAS), because these conventional approaches require complex procedures that usually need samples of hundreds or thousands of cases and a long time period, as well as being quite costly. In comparison to these approaches, ExpressGenotyping analysis can analyze the expression of each allele in a sample separately and, thus, reveal differences in allelic gene expression within the sample. Therefore, ExpressGenotyping analysis can be used to perform practical genetic biomarker discovery in samples with smaller numbers, at low cost. The basic functional principle of ExpressGenotyping is as follows. 1) Cell lines (HapMap lymphocytes) are treated with the drug of interest. 2) RNA and DNA are prepared from drug-treated and untreated cell lines. 3) Combined SNP typing/expression profiling on Affymetrix GeneChip microarrays is performed. 4) Analysis of expression profiles (drug-treated vs. vehicle-treated) and drug-induced EAI is then done. 5) Candidate genetic biomarkers for drug response prediction are identified. 6) Candidate genetic markers are validated with sample material from clinical studies. These technologies combined allow the genome-wide detection of variations in gene expression of individual alleles within one sample and eliminate the requirement for extensive inter-sample statistical analyses to identify a correlation between gene expression and genotype. In this report, the genetic biomarkers were identified by combining clinical data of IBD patients with AZA/5-ASA-induced EAI obtained by ExpressGenotyping analysis. Many SNPs in different genes were identified as candidate genetic biomarkers showing the relationship biologically (GOTERM analysis of DAVID). Of the genetic biomarkers related to the clinical 6-TGN blood concentration, SNPs in SLC38A9 were the most significant. This SLC38A9 gene might have an important role as a transporter for various drugs. Therefore, this SNP identified by ExpressGenotyping analysis might serve as a genetic biomarker for prediction of the 6-TGN blood concentration in patients on AZA/5-ASA.

These results show that ExpressGenotyping analysis is a useful way to identify individual differences in the incidence of drug response and ADRs, and it is expected to be a new method to accelerate the achievement of personalized medicine. In the future, the EAI EG method can be applied to the analysis of ex ante evaluation of immunosuppressive agents in the treatment of IBD, such as AZA.

## Supporting Information

Figure S1 Scatter plot analysis. In order to determine the experimental conditions for ExpressGenotyping analysis, scatter plot analysis was performed with the U133 Plus 2.0 Array. Data analysis was used to average signal values of *GM18940, GM18942, and GM18943 (*Sample ID in HapMap lymphocytes from Japanese individuals).
(TIFF)

Figure S2 30 different HapMap lymphocytes. The catalog ID indicates the database number for HapMap lymphocytes from Japanese subjects.
(DOCX)

Figure S3 Correlation analysis of SNPs and 6-TGN risk ratios in IBD patients. Criteria: r≥0.4, p-values<0.01.
(XLSX)

Figure S4 SNP genotypes of TPMT. TPMT 11 SNP genotypes were analyzed for 32 IBD patients.
(DOCX)

Figure S5 AZA/5-ASA-induced EAI detected with ExpressGenotyping analysis. Criteria: AZA/5-ASA-induced EAI≥2.
(XLSX)

Figure S6 AZA/5-ASA-induced EAI by AB heterozygous genotype analysis of ExpressGenotyping data. This list shows the AZA/5-ASA-induced EAI in more than one (AB genotype in >3 of 30) HapMap lymphocyte.
(DOCX)

## References

[1] Candy S, Wright J, Gerber M, Adams G, Gerig M, et al (1995) A controlled double blind study of azathioprine in the management of Crohn's disease. Gut 37: 674–678. 8549944 10.1136/gut.37.5.674 PMC1382873

[2] Fraser AG, Orchard TR, Jewell DP (2002) The efficacy of azathioprine for the treatment of inflammatory bowel disease: a 30 year review. Gut 50: 485–489. 11889067 10.1136/gut.50.4.485 PMC1773162

[3] Present DH, Korelitz BI, Wisch N, Glass JL, Sachar DB, et al (1980) Treatment of Crohn's disease with 6-mercaptopurine. A long-term, randomized, double-blind study. N Engl J Med 302: 981–987. 6102739 10.1056/NEJM198005013021801

[4] Pearson DC, May GR, Fick GH, Sutherland LR (1995) Azathioprine and 6-mercaptopurine in Crohn disease. A meta-analysis. Ann Intern Med 123: 132–142. 7778826 10.7326/0003-4819-123-2-199507150-00009

[5] Timmer A, McDonald JW, Macdonald JK (2007) Azathioprine and 6-mercaptopurine for maintenance of remission in ulcerative colitis. Cochrane Database Syst Rev CD000478. 17253451 10.1002/14651858.CD000478.pub2

[6] Cuffari C, Theoret Y, Latour S, Seidman G (1996) 6-Mercaptopurine metabolism in Crohn's disease: correlation with efficacy and toxicity. Gut 39: 401–406. 8949645 10.1136/gut.39.3.401 PMC1383347

[7] Osterman MT, Kundu R, Lichtenstein GR, Lewis JD (2006) Association of 6-thioguanine nucleotide levels and inflammatory bowel disease activity: a meta-analysis. Gastroenterology 130: 1047–1053. 16618398 10.1053/j.gastro.2006.01.046

[8] Cuffari C, Dassopoulos T, Turnbough L, Thompson RE, Bayless TM (2004) Thiopurine methyltransferase activity influences clinical response to azathioprine in inflammatory bowel disease. Clin Gastroenterol Hepatol 2: 410–417. 15118980 10.1016/s1542-3565(04)00127-2

[9] Dubinsky MC, Lamothe S, Yang HY, Targan SR, Sinnett D, et al (2000) Pharmacogenomics and metabolite measurement for 6-mercaptopurine therapy in inflammatory bowel disease. Gastroenterology 118: 705–713. 10734022 10.1016/s0016-5085(00)70140-5

[10] Wright S, Sanders DS, Lobo AJ, Lennard L (2004) Clinical significance of azathioprine active metabolite concentrations in inflammatory bowel disease. Gut 53: 1123–1128. 15247179 10.1136/gut.2003.032896 PMC1774135

[11] de Jong DJ, Derijks LJ, Naber AH, Hooymans PM, Mulder CJ (2003) Safety of thiopurines in the treatment of inflammatory bowel disease. Scand J Gastroenterol Suppl: 69–72. 10.1080/00855920310002726 14743886

[12] Jharap B, Seinen ML, de Boer NK, van Ginkel JR, Linskens RK, et al (2010) Thiopurine therapy in inflammatory bowel disease patients: analyses of two 8-year intercept cohorts. Inflamm Bowel Dis 16: 1541–1549. 20155846 10.1002/ibd.21221

[13] Schwab M, Schaffeler E, Marx C, Fischer C, Lang T, et al (2002) Azathioprine therapy and adverse drug reactions in patients with inflammatory bowel disease: impact of thiopurine S-methyltransferase polymorphism. Pharmacogenetics 12: 429–436. 12172211 10.1097/00008571-200208000-00003

[14] Lichtenstein GR, Abreu MT, Cohen R, Tremaine W (2006) American Gastroenterological Association Institute medical position statement on corticosteroids, immunomodulators, and infliximab in inflammatory bowel disease. Gastroenterology 130: 935–939. 16530531 10.1053/j.gastro.2006.01.047

[15] Takatsu N, Matsui T, Murakami Y, Ishihara H, Hisabe T, et al (2009) Adverse reactions to azathioprine cannot be predicted by thiopurine S-methyltransferase genotype in Japanese patients with inflammatory bowel disease. J Gastroenterol Hepatol 24: 1258–1264. 19682195 10.1111/j.1440-1746.2009.05917.x

[16] Hiratsuka M, Inoue T, Omori F, Agatsuma Y, Mizugaki M (2000) Genetic analysis of thiopurine methyltransferase polymorphism in a Japanese population. Mutat Res 448: 91–95. 10751626 10.1016/s0027-5107(00)00004-x

[17] Ban H, Andoh A, Tanaka A, Tsujikawa T, Sasaki M, et al (2008) Analysis of thiopurine S-methyltransferase genotypes in Japanese patients with inflammatory bowel disease. Intern Med 47: 1645–1648. 18827410 10.2169/internalmedicine.47.1268

[18] Uchiyama K, Nakamura M, Kubota T, Yamane T, Fujise K, et al (2009) Thiopurine S-methyltransferase and inosine triphosphate pyrophosphohydrolase genes in Japanese patients with inflammatory bowel disease in whom adverse drug reactions were induced by azathioprine/6-mercaptopurine treatment. J Gastroenterol 44: 197–203. 19214663 10.1007/s00535-008-2307-1

[19] Derijks LJ, Gilissen LP, Engels LG, Bos LP, Bus PJ, et al (2006) Pharmacokinetics of 6-thioguanine in patients with inflammatory bowel disease. Ther Drug Monit 28: 45–50. 16418693 10.1097/01.ftd.0000179839.71138.6d

[20] Haglund S, Taipalensuu J, Peterson C, Almer S (2008) IMPDH activity in thiopurine-treated patients with inflammatory bowel disease - relation to TPMT activity and metabolite concentrations. Br J Clin Pharmacol 65: 69–77. 17662091 10.1111/j.1365-2125.2007.02985.x PMC2291267

[21] Kumagai K, Hiyama K, Ishioka S, Sato H, Yamanishi Y, et al (2001) Allelotype frequency of the thiopurine methyltransferase (TPMT) gene in Japanese. Pharmacogenetics 11: 275–278. 11337944 10.1097/00008571-200104000-00012

[22] Kubota T, Nishida A, Takeuchi K, Iida T, Yokota H, et al (2004) Frequency distribution of thiopurine S-methyltransferase activity in red blood cells of a healthy Japanese population. Ther Drug Monit 26: 319–321. 15167635 10.1097/00007691-200406000-00017

[23] Pike MG, Franklin CL, Mays DC, Lipsky JJ, Lowry PW, et al (2001) Improved methods for determining the concentration of 6-thioguanine nucleotides and 6-methylmercaptopurine nucleotides in blood. J Chromatogr B Biomed Sci Appl 757: 1–9. 11419732 10.1016/s0378-4347(00)00513-2

[24] Knight JC, Keating BJ, Kwiatkowski DP (2004) Allele-specific repression of lymphotoxin-alpha by activated B cell factor-1. Nat Genet 36: 394–399. 15052269 10.1038/ng1331

[25] Knight JC (2004) Allele-specific gene expression uncovered. Trends Genet 20: 113–116. 15049300 10.1016/j.tig.2004.01.001

[26] Korn JM, Kuruvilla FG, McCarroll SA, Wysoker A, Nemesh J, et al (2008) Integrated genotype calling and association analysis of SNPs, common copy number polymorphisms and rare CNVs. Nat Genet 40: 1253–1260. 18776909 10.1038/ng.237 PMC2756534

[27] Midorikawa Y, Yamamoto S, Ishikawa S, Kamimura N, Igarashi H, et al (2006) Molecular karyotyping of human hepatocellular carcinoma using single-nucleotide polymorphism arrays. Oncogene 25: 5581–5590. 16785998 10.1038/sj.onc.1209537

[28] Ishikawa S, Komura D, Tsuji S, Nishimura K, Yamamoto S, et al (2005) Allelic dosage analysis with genotyping microarrays. Biochem Biophys Res Commun 333: 1309–1314. 15982637 10.1016/j.bbrc.2005.06.040

[29] Komura D, Shen F, Ishikawa S, Fitch KR, Chen W, et al (2006) Genome-wide detection of human copy number variations using high-density DNA oligonucleotide arrays. Genome Res 16: 1575–1584. 17122084 10.1101/gr.5629106 PMC1665641

[30] Krynetski EY, Schuetz JD, Galpin AJ, Pui CH, Relling MV, et al (1995) A single point mutation leading to loss of catalytic activity in human thiopurine S-methyltransferase. Proc Natl Acad Sci U S A 92: 949–953. 7862671 10.1073/pnas.92.4.949 PMC42614

[31] Tai HL, Krynetski EY, Schuetz EG, Yanishevski Y, Evans WE (1997) Enhanced proteolysis of thiopurine S-methyltransferase (TPMT) encoded by mutant alleles in humans (TPMT*3A, TPMT*2): mechanisms for the genetic polymorphism of TPMT activity. Proc Natl Acad Sci U S A 94: 6444–6449. 9177237 10.1073/pnas.94.12.6444 PMC21069

[32] Tai HL, Fessing MY, Bonten EJ, Yanishevsky Y, d'Azzo A, et al (1999) Enhanced proteasomal degradation of mutant human thiopurine S-methyltransferase (TPMT) in mammalian cells: mechanism for TPMT protein deficiency inherited by TPMT*2, TPMT*3A, TPMT*3B or TPMT*3C. Pharmacogenetics 9: 641–650. 10591545 10.1097/01213011-199910000-00011

[33] Szumlanski C, Otterness D, Her C, Lee D, Brandriff B, et al (1996) Thiopurine methyltransferase pharmacogenetics: human gene cloning and characterization of a common polymorphism. DNA Cell Biol 15: 17–30. 8561894 10.1089/dna.1996.15.17

[34] Salavaggione OE, Wang L, Wiepert M, Yee VC, Weinshilboum RM (2005) Thiopurine S-methyltransferase pharmacogenetics: variant allele functional and comparative genomics. Pharmacogenet Genomics 15: 801–815. 16220112 10.1097/01.fpc.0000174788.69991.6b

[35] Otterness DM, Szumlanski CL, Wood TC, Weinshilboum RM (1998) Human thiopurine methyltransferase pharmacogenetics. Kindred with a terminal exon splice junction mutation that results in loss of activity. J Clin Invest 101: 1036–1044. 9486974 10.1172/JCI1004 PMC508655

[36] Morales A, Salguti S, Miao CL, Lewis JD (2007) Relationship between 6-mercaptopurine dose and 6-thioguanine nucleotide levels in patients with inflammatory bowel disease. Inflamm Bowel Dis 13: 380–385. 17206711 10.1002/ibd.20028

[37] He L, Vasiliou K, Nebert DW (2009) Analysis and update of the human solute carrier (SLC) gene superfamily. Hum Genomics 3: 195–206. 19164095 10.1186/1479-7364-3-2-195 PMC2752037

[38] Hediger MA, Romero MF, Peng JB, Rolfs A, Takanaga H, et al (2004) The ABCs of solute carriers: physiological, pathological and therapeutic implications of human membrane transport proteinsIntroduction. Pflugers Arch 447: 465–468. 14624363 10.1007/s00424-003-1192-y

[39] Hundal HS, Taylor PM (2009) Amino acid transceptors: gate keepers of nutrient exchange and regulators of nutrient signaling. Am J Physiol Endocrinol Metab 296: E603–613. 19158318 10.1152/ajpendo.91002.2008 PMC2670634

[40] Mackenzie B, Erickson JD (2004) Sodium-coupled neutral amino acid (System N/A) transporters of the SLC38 gene family. Pflugers Arch 447: 784–795. 12845534 10.1007/s00424-003-1117-9

[41] Fredriksson R, Nordstrom KJ, Stephansson O, Hagglund MG, Schioth HB (2008) The solute carrier (SLC) complement of the human genome: phylogenetic classification reveals four major families. FEBS Lett 582: 3811–3816. 18948099 10.1016/j.febslet.2008.10.016

[42] Kusuhara H, Sugiyama Y (2009) In vitro-in vivo extrapolation of transporter-mediated clearance in the liver and kidney. Drug Metab Pharmacokinet 24: 37–52. 19252335 10.2133/dmpk.24.37

[43] Sundberg BE, Waag E, Jacobsson JA, Stephansson O, Rumaks J, et al (2008) The evolutionary history and tissue mapping of amino acid transporters belonging to solute carrier families SLC32, SLC36, and SLC38. J Mol Neurosci 35: 179–193. 18418736 10.1007/s12031-008-9046-x

[44] Varoqui H, Zhu H, Yao D, Ming H, Erickson JD (2000) Cloning and functional identification of a neuronal glutamine transporter. J Biol Chem 275: 4049–4054. 10660562 10.1074/jbc.275.6.4049

[45] Chaudhry FA, Schmitz D, Reimer RJ, Larsson P, Gray AT, et al (2002) Glutamine uptake by neurons: interaction of protons with system a transporters. J Neurosci 22: 62–72. 11756489 10.1523/JNEUROSCI.22-01-00062.2002 PMC6757603

[46] Yao D, Mackenzie B, Ming H, Varoqui H, Zhu H, et al (2000) A novel system A isoform mediating Na+/neutral amino acid cotransport. J Biol Chem 275: 22790–22797. 10811809 10.1074/jbc.M002965200

[47] Sugawara M, Nakanishi T, Fei YJ, Huang W, Ganapathy ME, et al (2000) Cloning of an amino acid transporter with functional characteristics and tissue expression pattern identical to that of system A. J Biol Chem 275: 16473–16477. 10747860 10.1074/jbc.C000205200

[48] Reimer RJ, Chaudhry FA, Gray AT, Edwards RH (2000) Amino acid transport system A resembles system N in sequence but differs in mechanism. Proc Natl Acad Sci U S A 97: 7715–7720. 10859363 10.1073/pnas.140152797 PMC16610

[49] Hatanaka T, Huang W, Ling R, Prasad PD, Sugawara M, et al (2001) Evidence for the transport of neutral as well as cationic amino acids by ATA3, a novel and liver-specific subtype of amino acid transport system A. Biochim Biophys Acta 1510: 10–17. 11342143 10.1016/s0005-2736(00)00390-4

[50] Sugawara M, Nakanishi T, Fei YJ, Martindale RG, Ganapathy ME, et al (2000) Structure and function of ATA3, a new subtype of amino acid transport system A, primarily expressed in the liver and skeletal muscle. Biochim Biophys Acta 1509: 7–13. 11118514 10.1016/s0005-2736(00)00349-7

[51] Desforges M, Lacey HA, Glazier JD, Greenwood SL, Mynett KJ, et al (2006) SNAT4 isoform of system A amino acid transporter is expressed in human placenta. Am J Physiol Cell Physiol 290: C305–312. 16148032 10.1152/ajpcell.00258.2005

[52] Chaudhry FA, Reimer RJ, Krizaj D, Barber D, Storm-Mathisen J, et al (1999) Molecular analysis of system N suggests novel physiological roles in nitrogen metabolism and synaptic transmission. Cell 99: 769–780. 10619430 10.1016/s0092-8674(00)81674-8

[53] Nakanishi T, Kekuda R, Fei YJ, Hatanaka T, Sugawara M, et al (2001) Cloning and functional characterization of a new subtype of the amino acid transport system N. Am J Physiol Cell Physiol 281: C1757–1768. 11698233 10.1152/ajpcell.2001.281.6.C1757

[54] Cubelos B, Gonzalez-Gonzalez IM, Gimenez C, Zafra F (2005) Amino acid transporter SNAT5 localizes to glial cells in the rat brain. Glia 49: 230–244. 15390093 10.1002/glia.20106

[55] Gu S, Roderick HL, Camacho P, Jiang JX (2000) Identification and characterization of an amino acid transporter expressed differentially in liver. Proc Natl Acad Sci U S A 97: 3230–3235. 10716701 10.1073/pnas.050318197 PMC16221

[56] Melone M, Quagliano F, Barbaresi P, Varoqui H, Erickson JD, et al (2004) Localization of the glutamine transporter SNAT1 in rat cerebral cortex and neighboring structures, with a note on its localization in human cortex. Cereb Cortex 14: 562–574. 15054072 10.1093/cercor/bhh018

[57] Gonzalez-Gonzalez IM, Cubelos B, Gimenez C, Zafra F (2005) Immunohistochemical localization of the amino acid transporter SNAT2 in the rat brain. Neuroscience 130: 61–73. 15561425 10.1016/j.neuroscience.2004.09.023

[58] Nakanishi T, Sugawara M, Huang W, Martindale RG, Leibach FH, et al (2001) Structure, function, and tissue expression pattern of human SN2, a subtype of the amino acid transport system N. Biochem Biophys Res Commun 281: 1343–1348. 11243884 10.1006/bbrc.2001.4504

