# Supplementary figures and images for: New Genetic Biomarkers Predicting Azathioprine Blood Concentrations in Combination Therapy with 5-Aminosalicylic Acid
Source: PLoS One. 2014 Apr 24;9(4):e95080. doi: 10.1371/journal.pone.0095080 (PMC3999094; doi:10.1371/journal.pone.0095080)

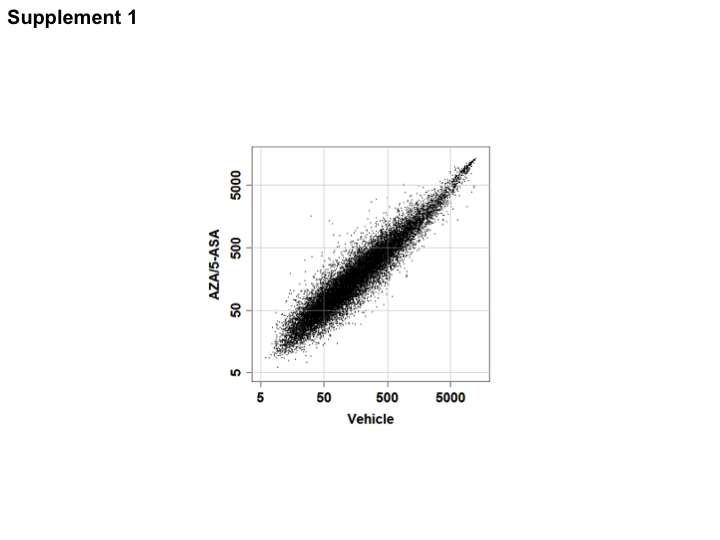

Supplement: Figure S1 — Scatter plot analysis. In order to determine the experimental conditions for ExpressGenotyping analysis, scatter plot analysis was performed with the U133 Plus 2.0 Array. Data analysis was used to average signal values of *GM18940, GM18942, and GM18943 (*Sample ID in HapMap lymphocytes from Japanese individuals). (TIFF) [file pone.0095080.s001.tif]
