# Supplementary material for: New Genetic Biomarkers Predicting Azathioprine Blood Concentrations in Combination Therapy with 5-Aminosalicylic Acid
Source: PLoS One. 2014 Apr 24;9(4):e95080. doi: 10.1371/journal.pone.0095080 (PMC3999094; doi:10.1371/journal.pone.0095080)
Supplement: Figure S2 — 30 different HapMap lymphocytes. The catalog ID indicates the database number for HapMap lymphocytes from Japanese subjects. (DOCX) [file pone.0095080.s002.docx]

Supplement 2

30 different HapMap lymphocytes of Japanese subjects used in this study

|  | Catalog ID | Gender |
| --- | --- | --- |
| 1 | GM18940 | Male |
| 2 | GM18942 | Female |
| 3 | GM18943 | Male |
| 4 | GM18944 | Male |
| 5 | GM18945 | Male |
| 6 | GM18947 | Female |
| 7 | GM18949 | Female |
| 8 | GM18951 | Female |
| 9 | GM18952 | Male |
| 10 | GM18953 | Male |
| 11 | GM18956 | Female |
| 12 | GM18959 | Male |
| 13 | GM18960 | Male |
| 14 | GM18961 | Male |
| 15 | GM18964 | Female |
| 16 | GM18965 | Male |
| 17 | GM18966 | Male |
| 18 | GM18967 | Male |
| 19 | GM18968 | Female |
| 20 | GM18969 | Female |
| 21 | GM18970 | Male |
| 22 | GM18971 | Male |
| 23 | GM18972 | Female |
| 24 | GM18973 | Female |
| 25 | GM18974 | Male |
| 26 | GM18975 | Female |
| 27 | GM18976 | Female |
| 28 | GM18978 | Female |
| 29 | GM18980 | Female |
| 30 | GM18981 | Female |
