# Supplementary material for: New Genetic Biomarkers Predicting Azathioprine Blood Concentrations in Combination Therapy with 5-Aminosalicylic Acid
Source: PLoS One. 2014 Apr 24;9(4):e95080. doi: 10.1371/journal.pone.0095080 (PMC3999094; doi:10.1371/journal.pone.0095080)
Supplement: Figure S4 — SNP genotypes of TPMT. TPMT 11 SNP genotypes were analyzed for 32 IBD patients. (DOCX) [file pone.0095080.s004.docx]

Supplement 4

Distribution of SNPs genotypes in TPMT

| SNPs | Genotypes | Number of SNP genotypes in TPMT for 32 IBD patients |
| --- | --- | --- |
|  |  |  |
| rs1142345 | AA | 32 |
|  | AG | - |
|  | GG | - |
| rs1800584 | AA | - |
|  | AG | - |
|  | GG | 32 |
| rs56161402 | CC | 32 |
|  | CT | - |
|  | TT | - |
| rs6921269 | CC | 32 |
|  | CT | - |
|  | TT | - |
| rs2842951 | CC | 22 |
|  | CT | 7 |
|  | TT | 3 |
| rs2842934 | AA | 22 |
|  | AG | 10 |
|  | GG | - |
| rs1800460 | AA | - |
|  | AG | - |
|  | GG | 32 |
| rs2518463 | CC | 4 |
|  | CT | 18 |
|  | TT | 10 |
| rs72552739 | AA | - |
|  | AC | - |
|  | CC | 32 |
| rs1800462 | CC | - |
|  | CG | - |
|  | GG | 32 |
| rs3898137 | AA | 4 |
|  | AG | 13 |
|  | GG | 15 |
