# Supplementary material for: New Genetic Biomarkers Predicting Azathioprine Blood Concentrations in Combination Therapy with 5-Aminosalicylic Acid
Source: PLoS One. 2014 Apr 24;9(4):e95080. doi: 10.1371/journal.pone.0095080 (PMC3999094; doi:10.1371/journal.pone.0095080)
Supplement: Figure S6 — AZA/5-ASA-induced EAI by AB heterozygous genotype analysis of ExpressGenotyping data. This list shows the AZA/5-ASA-induced EAI in more than one (AB genotype in >3 of 30) HapMap lymphocyte. (DOCX) [file pone.0095080.s006.docx]

Supplement 6

AZA/5-ASA-induced EAI by AB heterozygous genotype analysis

| SNPs | Chr | Genes | p-value | fold-change |
| --- | --- | --- | --- | --- |
| rs2129523 | 11 | GALNTL4(intron) | 3.E-08 | 3.14 |
| rs12433161 | 14 | NPAS3(intron) | 2.E-04 | 3.12 |
| rs6015561 | 20 | PHACTR3(intron) | 1.E-08 | 3.08 |
| rs8116068 | 20 | MACROD2(intron) | 2.E-03 | 3.03 |
| rs10827168 | 10 | C10orf112(intron) | 4.E-03 | 2.95 |
| rs11209805 | 1 | NEGR1(intron) | 8.E-03 | 2.63 |
| rs200956 | 6 | HIST1H2BF(intron) | 9.E-05 | 2.47 |
| rs2933134 | 3 | ATG3(intron) | 5.E-04 | 2.47 |
| rs13209042 | 6 | RPL30P8(intron) | 2.E-08 | 2.44 |
| rs4910353 | 11 | GALNTL4(intron) | 1.E-09 | 2.41 |
| rs1323067 | 4 | SLIT2(intron) | 7.E-04 | 2.41 |
| rs2240092 | 7 | COBL(intron) | 4.E-06 | 2.39 |
| rs9342639 | 6 | KHDRBS2(intron) | 7.E-03 | 2.34 |
| rs7559441 | 2 | RASGRP3(intron) | 1.E-07 | 2.33 |
| rs38589 | 7 | LOC442609(intron) | 4.E-03 | 2.30 |
| rs16903966 | 5 | EGFLAM(intron) | 3.E-08 | 2.27 |
| rs9394524 | 6 | GLO1(intron) | 5.E-07 | 2.27 |
| rs2198050 | 7 | TXNDC3(intron) | 6.E-05 | 2.25 |
| rs12959382 | 18 | FAU(CDS) | 1.E-03 | 2.23 |
| rs1032984 | 4 | APBB2(intron) | 3.E-04 | 2.21 |
| rs7934165 | 11 | BDNF(intron) | 3.E-05 | 2.21 |
| rs2614063 | 8 | LOC100129848(intron) | 3.E-06 | 2.21 |
| rs1182497 | 20 | PHACTR3(intron) | 4.E-04 | 2.20 |
| rs6982545 | 8 | TRAPPC9(intron) | 7.E-03 | 2.20 |
| rs11125786 | 2 | LOC647038(intron) | 3.E-11 | 2.19 |
| rs7073867 | 10 | ZNF365(intron) | 2.E-03 | 2.18 |
| rs9883580 | 3 | MAGI1(intron) | 5.E-12 | 2.17 |
| rs13306276 | 2 | COL3A1(intron) | 7.E-03 | 2.17 |
| rs2241172 | 17 | FLJ37644(intron) | 3.E-06 | 2.17 |
| rs6497523 | 16 | GRIN2A(intron) | 7.E-04 | 2.17 |
| rs11647877 | 16 | GRIN2A(intron) | 6.E-03 | 2.16 |
| rs1342514 | 1 | C1orf168(intron) | 1.E-03 | 2.13 |
| rs9656608 | 7 | ABCA13(intron) | 1.E-02 | 2.13 |
| rs12437038 | 14 | NPAS3(intron) | 6.E-04 | 2.12 |
| rs6730761 | 2 | C2orf39(intron) | 9.E-08 | 2.12 |
| rs6575209 | 14 | CATSPERB(intron) | 3.E-03 | 2.11 |
| rs2671385 | 11 | DYNC2H1(intron) | 3.E-05 | 2.10 |
| rs396491 | 16 | KIAA1609(intron) | 2.E-06 | 2.10 |
| rs631318 | 11 | ALG9(intron) | 3.E-05 | 2.09 |
| rs9311623 | 3 | ARHGEF3(intron) | 1.E-04 | 2.08 |
| rs17021897 | 2 | FAM82A1(intron) | 2.E-03 | 2.08 |
| rs887229 | 17 | TMEM132E(intron) | 8.E-08 | 2.08 |
| rs2429646 | 16 | A2BP1(intron) | 2.E-03 | 2.06 |
| rs1886779 | 14 | NPAS3(intron) | 7.E-05 | 2.05 |
| rs16824484 | 2 | LOC730124(intron) | 3.E-05 | 2.05 |
| rs2054492 | 14 | PELI2(intron) | 3.E-06 | 2.04 |
| rs7209210 | 17 | MAP2K6(intron) | 3.E-05 | 2.04 |
| rs7504263 | 18 | LOC100130480(intron) | 8.E-08 | 2.04 |
| rs2243267 | 5 | IL4(intron) | 3.E-06 | 2.04 |
| rs7692329 | 4 | SLIT2(intron) | 4.E-08 | 2.03 |
| rs267529 | 3 | ITGA9(intron) | 3.E-04 | 2.03 |
| rs4597720 | 3 | NAALADL2(intron) | 8.E-03 | 2.03 |
| rs950155 | 3 | ARHGEF3(intron) | 4.E-06 | 2.03 |
| rs7430096 | 3 | NAALADL2(intron) | 4.E-03 | 2.02 |
| rs944095 | 13 | FARP1(intron) | 4.E-06 | 2.02 |
| rs12901125 | 15 | LOC730126(intron) | 2.E-10 | 2.02 |
| rs2251036 | 21 | GRIK1(intron) | 4.E-03 | 2.02 |
| rs2241090 | 19 | IFI30(intron) | 5.E-03 | 2.02 |
| rs11613187 | 12 | TSPAN11(intron) | 3.E-04 | 2.02 |
| rs10737329 | 1 | LOC400796(intron) | 1.E-03 | 2.02 |
| rs11622592 | 14 | PELI2(intron) | 2.E-05 | 2.01 |
| rs2894129 | 10 | ADAMTS14(intron) | 4.E-04 | 2.00 |
| rs2297810 | 1 | CYP4B1(CDS) | 3.E-05 | 2.00 |
| rs2804262 | 9 | KANK1(intron) | 3.E-03 | 2.00 |
